# Leveraging Artificial Intelligence in Healthcare in Cambodia: Enhancing Access and Patient Outcomes

**DOI:** 10.1002/puh2.70433

**Published:** 2026-10-27

**Authors:** Sokchan Lorn, Yanvary Chhon, Techly San, Virak Sorn

**Affiliations:** ^1^ Faculty of Health Sciences and Biotechnology University of Puthisastra Phnom Penh Cambodia; ^2^ Faculty of Dentistry University of Puthisastra Phnom Penh Cambodia

**Keywords:** artificial intelligence, Cambodia, clinical decision support, digital health, health equity, health systems, healthcare, telemedicine

## Abstract

Artificial intelligence (AI) is increasingly reshaping healthcare globally and may offer opportunities to strengthen health systems in low‐ and middle‐income countries (LMICs), including Cambodia. AI‐enabled technologies, including machine learning, deep learning, predictive analytics, computer vision, wearable devices, and generative AI, could help address persistent health‐system challenges such as shortages and maldistribution of healthcare professionals, limited specialist services, unequal access to care, and constrained resources, particularly in rural and underserved communities. However, translating these technological opportunities into meaningful health benefits requires more than access to AI tools. Cambodia faces important barriers related to digital infrastructure, health‐information systems, data quality and availability, workforce capacity, cybersecurity, algorithmic bias, affordability, and regulatory and ethical governance. At the same time, ongoing digital transformation, the expansion of electronic health information systems, emerging AI research capacity, and national efforts toward AI governance provide an evolving foundation for responsible adoption. This perspective examines the current and potential role of AI in Cambodia's healthcare system, focusing on applications in disease diagnosis, clinical decision support, telemedicine, public health surveillance, healthcare administration, and health‐system management. Particular attention is given to the gap between technological feasibility and sustainable health‐system implementation, including local validation, interoperability, economic evaluation, workforce preparedness, and long‐term financing. AI should therefore be introduced through a phased, evidence‐based, and patient‐centered approach in which technology complements rather than replaces healthcare professionals. Investments in digital infrastructure, human capacity, locally relevant research, ethical governance, and sustainable implementation will be essential to ensure that AI contributes to improved healthcare access, quality, equity, and patient outcomes in Cambodia.

## Graphical abstract

This illustrates a proposed pathway for responsible AI integration into Cambodia's health system. The framework connects Cambodia's emerging digital‐health foundation and priority health needs with potential AI applications through a staged implementation pathway emphasizing data readiness, local validation, ethical and regulatory review, human oversight, pilot implementation, monitoring, and scale‐up. The framework highlights responsible AI governance—including safety, equity, privacy, transparency, and accountability—and identifies potential benefits in healthcare access, disease detection, clinical decision‐making, and health‐system efficiency. AI is positioned as a tool to augment healthcare professionals rather than replace them.

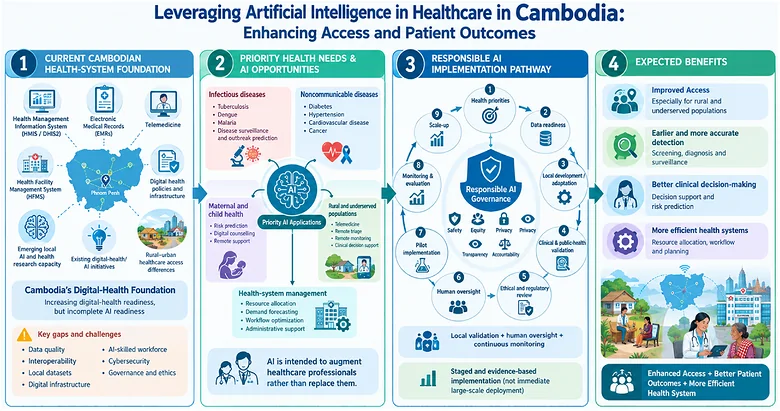

## 1 Introduction

Artificial intelligence (AI) has emerged as an important area of innovation in modern medicine and public health. Broadly, AI refers to computational systems capable of performing tasks that involve aspects of human intelligence, including learning, reasoning, prediction, pattern recognition, language processing, and decision‐making [1, 2]. Advances in machine learning, deep learning, computer vision, natural language processing, and generative AI have expanded the range of potential applications across healthcare and public health [3, 4, 5]. Although AI adoption is progressing rapidly in high‐income settings, its implementation in low‐ and middle‐income countries (LMICs), including Cambodia, remains comparatively limited and less well characterized.

AI is being explored across multiple areas of healthcare, including disease diagnosis, radiology, pathology, clinical decision support, precision medicine, drug discovery, public health surveillance, and health administration [2, 4, 5]. Existing evidence suggests that AI‐assisted systems may support earlier disease detection, diagnostic interpretation, treatment planning, risk prediction, and operational efficiency [6, 7, 8, 9]. Medical imaging is one of the most developed application areas, with deep learning and computer vision systems increasingly used to analyze radiological, ophthalmological, and histopathological images [10, 11, 12]. However, evidence of technical performance under controlled conditions does not necessarily translate into improved outcomes when these technologies are introduced into routine healthcare environments.

The COVID‐19 pandemic accelerated the adoption of digital health technologies and highlighted the importance of telemedicine, remote monitoring, and technology‐supported models of care [13, 14]. Digital health has subsequently gained greater attention as a means of addressing workforce shortages, geographic barriers, and continuity‐of‐care challenges in resource‐constrained settings [15, 16, 17]. AI may extend these capabilities through automated decision support, predictive analytics, intelligent triage, remote monitoring, and natural‐language interfaces. Nevertheless, AI should be understood as one component of a broader digital‐health ecosystem rather than as a substitute for foundational health‐system infrastructure.

Cambodia has made substantial progress in improving health outcomes over recent decades, but important health‐system challenges remain. These include limitations in health workforce capacity, the unequal distribution of healthcare professionals between urban and rural areas, shortages of specialist services, infrastructure constraints, and limited access to advanced diagnostic technologies [18, 19]. Rural populations may face additional barriers associated with distance, transportation costs, affordability, and limited availability of healthcare services, potentially contributing to delayed diagnosis and treatment [20, 21]. At the same time, Cambodia continues to manage communicable diseases and maternal and child health priorities while experiencing a growing burden of noncommunicable diseases (NCDs), including cardiovascular disease, diabetes, hypertension, and cancer [20, 21, 22]. These overlapping health needs place increasing demands on the health system and underscore the importance of approaches that can expand capacity without compromising quality or equity.

Against this background, AI‐enabled technologies may offer opportunities to strengthen healthcare delivery in Cambodia. Potential applications include AI‐assisted diagnosis, telemedicine, remote monitoring, clinical decision support, disease surveillance, and health‐system management [8, 21]. Digital‐health developments in Cambodia and neighboring countries suggest that technology can support remote care, chronic disease management, surveillance, and health‐system monitoring [21, 22, 23, 24]. However, the potential value of AI should not be inferred simply from technological availability. Its effectiveness will depend on the quality and representativeness of health data, interoperability of information systems, digital infrastructure, workforce capacity, affordability, cybersecurity, governance, and the ability to demonstrate meaningful benefits in local clinical and public health settings.

Cambodia is increasingly developing the policy and institutional foundations required for digital transformation and responsible AI adoption. Government agencies, academic institutions, and international partners have supported initiatives related to digital health, AI readiness, research capacity, and governance [24, 25, 26, 27]. Nevertheless, the country remains at an early stage of healthcare AI development, and evidence regarding its implementation, effectiveness, and impact on patient outcomes remains limited. This perspective therefore examines the current and potential role of AI in Cambodia's healthcare system, with particular attention to its potential contribution to healthcare access, clinical care, public health, and health‐system efficiency. It also considers the infrastructure, data, workforce, economic, ethical, and governance requirements that will determine whether AI can be implemented safely, equitably, and sustainably.

## 2 Methods

This perspective article adopted a narrative review approach to examine the opportunities, challenges, risks, ethical considerations, and implementation requirements associated with AI in healthcare, with particular emphasis on Cambodia and implications for LMIC health systems. The objective was to provide a contextual synthesis of the available evidence rather than a systematic assessment of the literature.

A literature search was conducted throughout August 2026 using Google Scholar and PubMed to identify relevant peer‐reviewed publications. Additional gray literature was reviewed from the websites of national and international organizations, including policy documents, digital‐health strategies, AI‐related initiatives, and health‐system reports relevant to Cambodia. Sources were selected based on their relevance to healthcare AI, digital‐health development, health‐system capacity, governance, and implementation in Cambodia or comparable LMIC settings.

As this was a narrative perspective rather than a systematic review, no formal systematic‐review protocol, meta‐analysis, risk‐of‐bias assessment, or quantitative synthesis was undertaken. The evidence was interpreted thematically to identify potential applications of AI, contextual opportunities and constraints, and priorities for responsible implementation in Cambodia.

## 3 AI in Healthcare: Opportunities and Preconditions

AI encompasses a broad group of computational approaches capable of performing tasks that involve learning, prediction, pattern recognition, language processing, and decision‐making. Healthcare applications include machine learning, deep learning, computer vision, natural language processing, predictive analytics, robotics, and, increasingly, generative AI and large language models [1, 2, 3].

Machine learning algorithms identify patterns within data to generate predictions or classifications, whereas deep learning uses multilayer neural networks to process complex information, including medical images and clinical records [5]. Computer vision enables automated interpretation of visual information, whereas natural language processing can extract and interpret information from clinical documentation and other unstructured text. More recently, generative AI and large language models have expanded the potential use of AI in healthcare education, clinical documentation, patient communication, research, administration, and decision support [23, 28, 29, 30]. These applications remain subject to important limitations, including inaccurate outputs, misinformation, limited explainability, privacy risks, and uncertain accountability.

Across healthcare, AI has demonstrated potential in diagnostic imaging, risk prediction, clinical decision support, disease surveillance, remote monitoring, and health‐system management [6, 7, 8, 9, 10, 11, 12, 31, 32, 33, 34, 35, 36, 37, 38, 39, 40, 41, 42, 43]. AI‐assisted imaging, for example, may support the detection of tuberculosis, breast cancer, diabetic retinopathy, and cardiovascular disease [10, 11]. In oncology, AI is being investigated for tumor detection, risk stratification, treatment planning, and precision medicine [12]. Predictive models can potentially identify patients at increased risk of disease or adverse outcomes and support the allocation of healthcare resources [32]. AI may also complement telemedicine and wearable technologies by supporting automated monitoring, triage, and risk identification.

However, the clinical and public health value of AI depends on factors beyond algorithmic performance. Common limitations in healthcare AI research include overfitting, inadequate external validation, poor reproducibility, algorithmic bias, limited transparency, privacy concerns, and insufficient evaluation under real‐world conditions [14, 15, 16, 30, 31, 32, 33]. An algorithm that performs well on a development dataset may perform differently when applied to another population, healthcare facility, disease prevalence pattern, or technological environment. Consequently, responsible AI implementation requires a broader evaluation framework encompassing technical performance, clinical effectiveness, usability, equity, safety, cost, interoperability, and sustainability.

For Cambodia, this distinction is particularly important. The central question is not simply whether AI technologies are technically available, but whether they can be reliably integrated into the country's health system and generate measurable benefits for patients and populations. This requires an appropriate digital foundation, representative data, trained personnel, governance mechanisms, local validation, sustainable financing, and integration with existing healthcare workflows.

### 3.1 Cambodia's Digital Health and AI Readiness

Cambodia has made important progress in developing the digital foundations that could support future healthcare AI. On 15 May 2026, the Ministry of Health (MOH), in collaboration with WHO, UNICEF, the Clinton Health Access Initiative, and Gavi, launched a new electronic Health Management Information System (HMIS) based on the District Health Information Software 2 (DHIS2) platform, with nationwide implementation across all 25 provinces [34, 35, 36]. The system is designed to support health service and disease reporting, data visualization, monitoring, and evidence‐informed decision‐making. Cambodia has also continued to develop electronic medical record (EMR) systems as part of broader health‐sector digital transformation and efforts toward universal health coverage [35].

These developments are important because AI depends on the availability of structured and accessible data. However, digital infrastructure should not be equated with AI readiness. Electronic reporting systems can create the foundation for data‐driven healthcare, but effective AI implementation additionally requires data quality, interoperability, appropriate governance, computational capacity, cybersecurity, skilled personnel, and mechanisms for clinical validation [36].

The Cambodia Digital Economy and Society Policy Framework 2021–2035 provides a broader policy direction for national digital transformation, emphasizing digital infrastructure, adoption, digital government, human‐resource development, data, innovation, cybersecurity, and trust [36, 37]. These priorities are directly relevant to healthcare AI because reliable connectivity, interoperable information systems, secure data environments, and skilled personnel are prerequisites for responsible implementation. Similarly, the Cambodia Digital Government Policy 2022–2035 provides an enabling framework for integrated digital services and improved government information systems, including health‐related systems [27]. Integration is particularly important in healthcare because AI applications often require data from multiple sources, including clinical records, laboratory systems, imaging, pharmacy systems, and public health surveillance platforms. Without interoperability, AI applications may remain isolated within individual projects or facilities and have limited value at the health‐system level.

Cambodia is also expanding digital service delivery through health facility information systems and telemedicine initiatives. The MOH has reported progress in developing a Health Facility Management System (HFMS), including dashboards containing information on private health facilities, whereas telemedicine has been planned for pilot implementation in selected hospitals [38]. These developments could eventually support AI‐enhanced consultation, triage, monitoring, and resource management. Nevertheless, their current significance lies primarily in strengthening the digital infrastructure required for future AI applications rather than demonstrating mature clinical AI deployment.

Institutional capacity for health‐related AI research is also emerging. On 28 January 2026, the Cambodia Academy of Digital Technology and the University of Health Sciences established a collaboration focused on applied AI research and development in health [39]. The collaboration includes capacity building in data science and computing, ethically managed health data, potential health applications, and educational development. Such partnerships could contribute to a locally relevant evidence base and strengthen the country's ability to develop, validate, and evaluate AI technologies in Cambodian settings. Table 1 therefore summarizes the relationship between Cambodia's existing digital‐health capabilities, remaining system gaps, AI prerequisites, and implications for future healthcare AI implementation.

**TABLE 1:** Cambodia's current digital‐health capabilities and artificial intelligence (AI) prerequisites.

Cambodia's current capability	Current situation in Cambodia	AI prerequisite	Implication for AI adoption
National Health Management Information System (HMIS)	Cambodia launched a new electronic HMIS based on DHIS2 in May 2026, with nationwide implementation across all 25 provinces The system supports health service and disease reporting, dashboards, data visualization, monitoring, planning, and decision‐making [34]	Timely, standardized, interoperable, and sufficiently complete health data [16, 30, 42]	Provides an important digital foundation for future AI‐supported surveillance, prediction, planning, and resource allocation, but HMIS availability alone does not constitute AI readiness [16, 30, 34, 42]
Electronic medical records (EMRs)	Cambodia has been advancing the development of an electronic medical record management system as part of broader health‐system digital transformation and universal health coverage efforts [35]	Structured longitudinal patient data, interoperability, standardized data elements, privacy protection, cybersecurity, and appropriate access controls [16, 30, 42]	EMRs could provide important data for clinical decision support, risk prediction, medication safety, and personalized care, provided data quality and interoperability are established [16, 30, 42]
Telemedicine	Cambodia has been developing digital health services, including plans for telemedicine pilot implementation in selected hospitals [36]	Reliable connectivity, secure data exchange, digital literacy, clinical workflows, human oversight, and clear referral/escalation mechanisms [7, 13, 16, 20]	Provides a potential platform for future AI‐supported remote consultation, triage, monitoring, and patient communication [7, 9, 13, 36]
Health Facility Management System (HFMS)	The Ministry of Health has developed an HFMS and associated dashboard to support information on health facilities and health‐system management [36]	Integrated facility‐level data, standardized reporting, interoperability, data quality, and appropriate governance [16, 30, 42]	Could support AI applications for service planning, resource allocation, facility management, and demand prediction [30, 34, 42]
Digital‐health policy environment	Cambodia's Digital Economy and Society Policy Framework 2021–2035 establishes national priorities for digital infrastructure, digital government, human resources, digital transformation, and trust/reliability [26, 27]	Sustained infrastructure investment, institutional capacity, interoperability, cybersecurity, and governance [26, 42]	Provides a broader policy foundation for digital‐health development and future AI integration [26, 42]
Digital government and health information systems	The Cambodia Digital Government Policy 2022–2035 promotes integrated digital government services and information systems, including health‐related digital transformation [37]	Interoperable government and health information systems, digital identity, secure data exchange, and governance [37]	Can facilitate cross‐sectoral data integration and digital infrastructure required for population‐level AI applications [35, 42]
National AI policy	Cambodia has been developing the Draft National Artificial Intelligence Strategy 2025–2030, with consultations involving government ministries and other stakeholders [27, 38]	Clear AI governance, ethical standards, accountability, regulatory mechanisms, and sector‐specific implementation guidance [27, 42]	Creates an emerging national policy basis for responsible AI adoption, although the strategy should not be described as fully implemented if referring to the documented draft/consultation stage [27, 38]
AI readiness assessment	Cambodia completed a national AI readiness assessment with UNESCO in 2025 to identify progress, opportunities, challenges, and recommendations relevant to national AI development [42]	Strengthened infrastructure, skills, data governance, research capacity, ethical safeguards, and institutional readiness [42, 43]	Provides a framework for identifying and addressing national AI‐readiness gaps before large‐scale healthcare implementation [42, 43]
Local health‐AI research capacity	Collaboration between Cambodian health and technology institutions is emerging, including applied AI research and development in health and related training initiatives [39]	Local multidisciplinary research teams, ethically managed datasets, computing capacity, clinical expertise, and research funding [16, 30, 42]	Can strengthen locally relevant AI development, validation, evaluation, and workforce capacity [16, 30, 39, 42]
Existing AI/digital‐health applications	Cambodia has experience with digital and AI‐related health initiatives, including i‐MoMCARE for maternal and child health and digital malaria surveillance [21, 40]	Local validation, scalability, interoperability, monitoring, sustainability, and integration into routine health services [16, 21, 42]	Demonstrates initial experience while highlighting the need to move from individual projects toward interoperable, evidence‐based, and scalable national systems [16, 21, 42, 43]
Rural digital access and health disparities	Rural populations may experience greater barriers to healthcare access, whereas digital‐health implementation can also be affected by connectivity, infrastructure, and digital‐literacy differences [7, 20]	Reliable connectivity, affordable devices, digital literacy, local‐language interfaces, and inclusive service design [7, 13, 20]	AI adoption should prioritize equity so that digital technologies do not widen existing rural–urban healthcare disparities [7, 20]
Data governance, cybersecurity, and ethics	Cambodia's AI‐readiness and digital‐transformation efforts highlight the importance of governance, trust, data, skills, and responsible technology adoption [26, 27, 42]	Privacy, cybersecurity, informed consent, transparency, explainability, accountability, and appropriate data governance [44, 45, 46]	These safeguards should be incorporated from the design stage rather than added after AI systems are deployed [45, 46]
AI‐skilled healthcare workforce	Cambodia's digital and AI transformation requires the development of relevant human resources and technical capacities [26, 27, 42]	Training in AI literacy, digital health, data science, clinical AI evaluation, ethics, and human–AI collaboration [26, 27, 42]	Workforce development is necessary for appropriate implementation, interpretation, oversight, and monitoring of AI systems [16, 30, 42]

Abbreviation: DHIS2, District Health Information Software 2.

### 3.2 Emerging AI Governance and National Readiness

Cambodia is also developing an emerging policy environment for AI governance. The Ministry of Post and Telecommunications led the preparation of the Draft National Artificial Intelligence Strategy 2025–2030 and conducted consultations with ministries and other stakeholders [27]. The MOH was specifically consulted in May 2025 regarding health‐sector priorities and potential applications of AI. The strategy‐development process included technical workshops, interministerial consultations, stakeholder engagement, and national discussions [40, 41]. Accordingly, this framework is more appropriately described as an emerging or draft policy direction rather than a fully implemented national AI strategy.

Cambodia's National AI Readiness Assessment, completed in 2025 in collaboration with UNESCO, provides further information regarding national preparedness for AI adoption [42]. More than 300 stakeholders from government and other sectors participated in the readiness process [43]. Importantly, AI readiness encompasses more than technical capability. Infrastructure, workforce skills, data availability, institutional capacity, governance, ethics, and public trust all influence whether AI can be implemented responsibly. Taken together, Cambodia is developing several important prerequisites for healthcare AI, including national digital‐health infrastructure expanding, EMR and telemedicine systems developing, digital governance evolving, institutional research capacity emerging, and a national AI policy framework being developed [34, 35, 36, 37, 38, 39, 40, 41, 42, 43]. However, significant gaps remain in data interoperability and quality, availability of representative local datasets, workforce capacity, cybersecurity, ethical governance, and clinical validation [36, 37, 42].

The implications are important. Cambodia should not approach AI adoption primarily as a technology acquisition process. Instead, AI development should proceed as part of a broader health‐system transformation in which digital foundations, local evidence generation, workforce development, governance, clinical validation, economic assessment, and implementation capacity develop together.

## 4 Potential Applications of AI in Cambodian Healthcare

### 4.1 AI for Disease Diagnosis and Medical Imaging

AI‐assisted diagnostic technologies have the potential to improve disease detection and diagnostic accuracy in Cambodia's healthcare system. Deep learning and computer vision have demoAI‐supported imaging tools that could assist healthcare professionals in detecting conditions such as tuberculosis, pneumonia, breast cancer, diabetic retinopathy, and cardiovascular disease [10, 11, 12], particularly in settings where specialist radiologists and other diagnostic experts are limited. AI‐assisted tuberculosis screening may be particularly relevant because tuberculosis remains an important public health concern in Cambodia and the wider Southeast Asian region.

AI may also strengthen pathology services through the automated analysis of histopathological images and the identification of abnormal or cancerous tissues. Such technologies could help reduce diagnostic delays and improve access to specialist diagnostic services, especially in underserved areas. In resource‐limited settings where trained specialists are scarce, AI‐based diagnostic systems should primarily be considered supportive tools that assist healthcare professionals rather than replace clinical judgment. Several studies have reported high diagnostic performance of AI‐assisted imaging systems under controlled research conditions [11, 12, 15]. However, performance in routine clinical practice may differ because of variations in data quality, healthcare infrastructure, patient populations, imaging equipment, and clinical workflows. Therefore, rigorous local validation and careful implementation are essential before these technologies are adopted on a large scale.

For Cambodia, local validation is particularly important. AI models developed using datasets from high‐income countries may not perform equally well in the Cambodian population because of differences in disease prevalence, demographic characteristics, imaging equipment, referral patterns, clinical practices, and data quality. Prospective studies should therefore evaluate AI systems under real‐world Cambodian conditions before wider deployment. Ongoing monitoring should also assess whether AI improves diagnostic access and performance while avoiding new disparities between urban and rural populations. Such an approach would help ensure that AI‐supported diagnosis contributes to equitable, safe, and context‐appropriate healthcare in Cambodia.

### 4.2 AI‐Enhanced Telemedicine and Remote Healthcare

Telemedicine may help address geographic barriers to healthcare access in Cambodia, particularly for rural and underserved populations. AI could extend telemedicine through automated symptom assessment, virtual triage, decision support, patient education, appointment management, and chronic disease monitoring [6, 13]. These functions could complement rather than replace clinician‐led remote consultations. AI‐powered chatbots and virtual assistants may support routine communication, medication reminders, appointment scheduling, and preliminary health information [34, 35]. Mobile applications and wearable devices could additionally support monitoring of parameters such as blood pressure, heart rate, glucose, sleep, and physical activity [18, 47]. For chronic diseases such as diabetes and hypertension, continuous or intermittent remote monitoring may facilitate earlier identification of abnormal patterns and more timely clinical review.

Cambodia's development of telemedicine provides a potential platform for these applications. The MOH has reported plans to pilot telemedicine services in selected hospitals to improve remote consultation and treatment [43]. However, the existence of telemedicine infrastructure should not be interpreted as evidence of established AI‐enabled remote care. Additional validation, workflow integration, data protection, and evaluation would be required before AI‐supported telemedicine could be implemented at scale. Equity must remain central to this development. Limited connectivity, affordability, digital literacy, access to smartphones, and differences in language or health literacy may prevent some populations from benefiting from digitally mediated care. AI‐enhanced telemedicine should therefore be evaluated according to whether it actually expands access among underserved populations rather than simply increasing the availability of technology for those who are already digitally connected.

### 4.3 AI for Public Health Surveillance and Epidemiology

AI could strengthen public health surveillance by integrating large and heterogeneous datasets, identifying temporal and spatial patterns, detecting unusual signals, and supporting forecasting and early warning activities [17, 45, 48]. Such applications may be relevant to Cambodia, where communicable diseases remain important while the burden of NCDs is increasing [18, 19, 20, 21, 22]. Cambodia has recently strengthened the digital infrastructure required for more advanced surveillance and analytics. In May 2026, the MOH, together with WHO, UNICEF, the Clinton Health Access Initiative, and Gavi, launched a DHIS2‐based electronic HMIS for nationwide implementation across all 25 provinces [34]. The system supports real‐time reporting, dashboards, visualization, and health‐system monitoring. Importantly, the HMIS is a digital‐health platform rather than an AI system. Its significance for future AI lies in its potential to provide more standardized and timely data for advanced analytics, forecasting, anomaly detection, and population‐level risk assessment.

Disease‐specific platforms provide additional examples. Cambodia's digital malaria information system integrates case‐based reporting, geographic information, dashboards, risk stratification, and public health response functions [49]. A 2026 implementation study reported use of the health‐center application across the malaria surveillance network to support case notification, classification, reactive case detection, and targeted responses [49]. Although not itself an AI system, this structured and geographically linked data environment provides a potential foundation for future predictive applications. Another relevant example is the Electronic clinical Decision support for Acute fever Management (EDAM) application implemented in primary health centers in rural Battambang Province [36, 37, 38]. EDAM uses a rule‐based algorithm incorporating clinical symptoms, vital signs, and point‐of‐care malaria and C‐reactive protein testing to support management of acute febrile illness [36, 39]. A cluster‐randomized trial involving 30 primary health centers demonstrated Cambodia's capacity to implement and evaluate algorithm‐supported decision tools in routine rural primary care [40]. However, the 2026 trial did not identify a statistically significant reduction in overall antibiotic prescribing, and adherence to recommendations was limited [41]. These findings highlight an important implementation lesson: algorithmic capability alone does not guarantee health‐system impact. User engagement, training, workflow compatibility, local adaptation, and organizational support are equally important. EDAM also illustrates the distinction between rule‐based digital decision support and AI. Its predefined logic does not constitute a machine‐learning system [36, 37, 38, 39]. Nevertheless, its integration of clinical and point‐of‐care information demonstrates the type of structured data environment that may support more advanced predictive approaches in the future.

EMRs and health‐facility information systems may provide additional longitudinal and facility‐level data for future analysis [35, 36]. If appropriately interoperable and governed, these data could support disease‐risk modeling, service‐demand forecasting, and epidemiological monitoring. Collaboration between the Cambodian Academy of Digital Technology and the University of Health Sciences may further strengthen local capacity to develop and evaluate such applications [38]. Future AI‐enabled surveillance could potentially combine HMIS data with laboratory results, EMRs, environmental information, demographic characteristics, climate variables, and other relevant datasets to identify emerging risks [17, 45, 48]. Potential applications include forecasting vector‐borne disease outbreaks, identifying geographical hotspots, anticipating healthcare demand, and supporting allocation of public health resources. For NCDs, integrated data could support population‐level risk stratification for conditions such as diabetes, hypertension, and cardiovascular disease.

However, increased data availability does not automatically justify AI‐based surveillance. Predictive models require representative, interoperable, continuously updated data and must be evaluated for changes in disease patterns and data quality [16, 30, 33, 45]. Privacy, cybersecurity, transparency, accountability, and algorithmic bias must also be addressed [44, 45, 46, 47, 48, 49, 50]. AI‐generated signals should complement, rather than replace, epidemiological expertise because automated systems can produce false alarms, miss emerging events, or perform differently across populations.

Overall, Cambodia is moving from conventional reporting toward increasingly digital and data‐driven surveillance. The DHIS2‐based HMIS, disease‐specific platforms, rule‐based decision‐support systems, developing EMRs, and emerging AI research capacity provide complementary building blocks for future AI‐supported epidemiology [27, 34, 35, 36, 38, 39, 49]. A logical next step is therefore not immediate large‐scale AI deployment but progressive development and prospective evaluation of locally relevant predictive applications under strong human oversight and governance.

### 4.4 AI for Clinical Decision Support

AI‐supported clinical decision systems may assist healthcare professionals with diagnosis, risk assessment, medication management, and treatment planning [31]. In Cambodia, such tools could be particularly relevant in primary care and facilities where access to specialist consultation is limited [42, 43, 50]. AI may help clinicians synthesize complex clinical information and identify patients who require further investigation or referral. However, clinical decision support presents a higher safety threshold than many administrative applications. Inaccurate or poorly calibrated recommendations may directly affect patient care. AI‐generated outputs should therefore remain advisory, with clinicians retaining responsibility for interpreting recommendations and making final decisions. Local validation, transparent performance monitoring, appropriate escalation pathways, and clear allocation of clinical responsibility will be essential.

### 4.5 AI for Healthcare Administration and Resource Management

AI may also support healthcare administration and operational efficiency through applications such as appointment scheduling, patient‐flow management, inventory forecasting, workforce planning, resource allocation, and workflow automation [51, 52]. Predictive analytics could help facilities anticipate patient demand, staffing requirements, and supply needs, whereas automation could reduce repetitive administrative tasks. In a resource‐constrained health system, such applications may be particularly valuable if they release staff time and improve the use of limited resources [51, 52, 53]. However, their implementation should be guided by measurable operational benefits and economic evaluation. Administrative AI should complement broader investments in essential services rather than divert resources from foundational health‐system needs.

Taken together, the potential applications of AI in Cambodia extend across the continuum from population‐level surveillance to individual clinical care and health‐system management. Table 2 summarizes selected priority applications in relation to disease burdens, health‐system needs, current gaps, and implementation considerations. The feasibility of these applications is likely to differ substantially. Use cases with clear health‐system value, available data, manageable implementation requirements, and opportunities for local validation may be more suitable for early evaluation than applications requiring highly sophisticated infrastructure or extensive proprietary datasets. AI adoption should therefore be guided by health need, evidence, feasibility, equity, affordability, and sustainability, rather than technological novelty alone.

**TABLE 2:** Priority artificial intelligence (AI) applications mapped to Cambodia's health‐system needs.

Health priority/system need	Potential AI application	Cambodia‐specific rationale/current gap	Key implementation consideration
Tuberculosis (TB)	AI‐assisted chest x‐ray interpretation, screening, and case prioritization	AI‐based medical‐image analysis may support TB screening and diagnosis, particularly where access to specialist interpretation is limited [2, 53, 54, 55]	Local validation, image quality, referral pathways, human oversight, and integration into existing TB services are required [16, 30, 33, 54]
Dengue and other infectious diseases	Predictive surveillance, outbreak detection, and risk mapping	Cambodia faces substantial infectious‐disease surveillance needs, creating opportunities for data‐driven prediction and early warning [17, 49]	Requires timely surveillance data, interoperability, epidemiological validation, and mechanisms for public‐health response [17, 48, 49]
Malaria	Real‐time surveillance, prediction, and spatial risk assessment	Cambodia has already demonstrated digital surveillance capacity through real‐time malaria surveillance initiatives [49]	AI should strengthen—not replace—existing surveillance and response systems, with attention to data quality and sustainability [16, 45, 48]
Diabetes	Risk prediction, retinal screening, self‐management support, and remote monitoring	Diabetes is an increasing noncommunicable‐disease concern, whereas digital self‐care interventions are emerging in Cambodia [22, 53]	Algorithms should be locally validated and linked with screening, treatment, and follow‐up services [10, 16, 53]
Hypertension and cardiovascular disease	Risk prediction, remote monitoring, and clinical decision support	Hypertension and cardiovascular disease require long‐term monitoring and risk management, creating opportunities for digital and AI‐supported care [22, 54]	Reliable patient data, device validation, clinical integration, and continuous monitoring are essential [9, 16, 22]
Cancer	AI‐assisted imaging, pathology, screening, and clinical decision support	AI has demonstrated potential in cancer detection and clinical oncology, which may be particularly relevant where specialist capacity is constrained [11, 12, 54]	Local validation, specialist oversight, diagnostic accuracy, and equitable access must be addressed [11, 12, 14, 33]
Maternal and child health (MCH)	Risk prediction, digital counseling, triage, and remote support	Cambodia has experience with digital maternal‐health interventions, including i‐MoMCARE [21]	AI should be integrated with existing maternal‐health workflows and validated for local populations and languages [16, 21, 27]
Rural and remote healthcare	AI‐supported telemedicine, triage, decision support, and remote monitoring	Rural populations face healthcare‐access disparities, whereas digital‐health services may help extend care to underserved areas [7, 20]	Connectivity, affordability, digital literacy, local‐language interfaces, and human referral pathways are critical [7, 13, 20]
Health workforce shortages	Clinical decision support, automated documentation, triage, and task support	AI may augment healthcare professionals by supporting selected diagnostics and administrative and information‐processing tasks [1, 28, 30]	AI should support rather than replace health professionals with appropriate training. accountability and oversight [1, 16, 30, 45]
Hospital and health‐facility management	Demand forecasting, resource allocation, workflow optimization, and administrative automation	Digital health‐facility management systems provide a potential foundation for future data‐driven optimization [51, 52, 53]	Interoperability, data quality, cybersecurity, and integration with routine management systems are required [16, 51, 52]
Public health planning and resource allocation	Predictive analytics, population‐risk modeling, and decision support	National health information infrastructure can provide a foundation for more data‐driven planning and monitoring [18, 19, 34]	AI outputs should be transparent, validated, interpretable, and incorporated into accountable decision‐making processes [16, 34, 45, 55, 56, 57, 58, 59]

## 5 Challenges and Ethical Considerations

The potential benefits of AI must be considered alongside substantial implementation challenges. In Cambodia, these challenges span infrastructure, data, workforce capacity, economics, governance, ethics, and long‐term health‐system integration.

### 5.1 Infrastructure and Interoperability

Digital infrastructure remains an important prerequisite, particularly in rural and remote areas where connectivity, electricity, hardware, and technical support may be limited. Continued reliance on paper‐based documentation in some settings may further complicate integration with AI‐enabled systems [60]. Fragmented information systems and limited interoperability can also prevent data from being used effectively across facilities and levels of care [61]. These constraints highlight a fundamental distinction between AI feasibility and AI sustainability. A technology may function successfully in a controlled pilot while remaining unsuitable for routine implementation if the underlying health system cannot support connectivity, data exchange, maintenance, and technical assistance.

### 5.2 The Risk of Pilot Project Syndrome

A related concern is “pilot project syndrome,” whereby promising digital interventions demonstrate feasibility on a limited scale but fail to achieve sustained integration into routine health services. In resource‐constrained settings, projects may depend on short‐term external financing, project‐specific infrastructure, or donor‐supported technical expertise. Fragmentation, weak coordination, limited interoperability, and insufficient local ownership can further constrain scale‐up [47, 48, 49, 50, 51, 52]. For Cambodia, the implications extend beyond AI. New AI initiatives should be designed from the outset around existing health‐system architecture, institutional ownership, national standards, workforce requirements, recurrent financing, and long‐term maintenance. Pilot projects should include explicit criteria for continuation, scale‐up, adaptation, or discontinuation rather than being treated as successful solely because technical feasibility has been demonstrated.

### 5.3 Data Quality, Representativeness, and Algorithmic Performance

AI systems depend on data that are sufficiently accurate, representative, structured, and relevant to the intended population. Fragmented or incomplete clinical data may reduce model reliability, whereas datasets derived predominantly from high‐income countries may not adequately reflect Cambodian populations or healthcare environments [14, 15, 16, 23, 33]. Differences in disease prevalence, demographic characteristics, clinical workflows, equipment, referral patterns, and documentation practices can affect algorithmic performance. Consequently, external evidence should be treated as informative but not automatically transferable to Cambodia. Local and prospective validation should assess performance across relevant populations and healthcare settings, with continued monitoring after deployment.

### 5.4 Economic Sustainability and Opportunity Costs

Economic sustainability is a central consideration because AI implementation involves both initial and recurrent expenditure. Costs may include hardware, connectivity, software development or licensing, cloud or server infrastructure, cybersecurity, data management, system integration, maintenance, technical support, workforce training, and periodic upgrades. The potential efficiency gains from AI should therefore not be assumed to represent economic value. Technologies must be considered in relation to competing health‐system priorities, including essential medicines, workforce expansion, primary healthcare infrastructure, diagnostic capacity, and other interventions that may produce substantial health benefits [62, 63]. Cost‐effectiveness analysis, budget‐impact assessment, and opportunity‐cost analysis under Cambodian conditions could help identify applications that provide sufficient value relative to their financial and organizational requirements.

### 5.5 Ethical, Legal, and Patient‐Safety Considerations

Healthcare AI involves sensitive personal information and therefore raises concerns regarding privacy, cybersecurity, informed consent, transparency, accountability, and data governance [44, 45, 46]. Cybersecurity weaknesses may expose patients to unauthorized access or data breaches. Algorithmic bias may result in systematically poorer performance for certain populations when training data are incomplete or unrepresentative.

Explainability is also important, particularly for clinical applications. Highly complex models may provide limited insight into how a recommendation was generated, potentially complicating clinician oversight and accountability [57]. In high‐risk applications such as diagnosis, triage, or treatment recommendations, appropriate safeguards are necessary to prevent inappropriate reliance on automated outputs. Clear governance should therefore define who is responsible for AI‐supported decisions, how systems are validated and approved, how adverse events are monitored, and what processes exist for auditing and updating models.

### 5.6 Workforce and Institutional Capacity

AI adoption also requires a workforce capable of using, evaluating, and governing these technologies. Healthcare professionals may require training in digital health, AI literacy, data interpretation, limitations of algorithmic systems, and responsible use. Educational institutions may therefore need to incorporate AI, health informatics, data science, ethics, and digital‐health competencies into health‐professions education [26, 27]. At the same time, AI should not be considered a substitute for investment in human resources. Where shortages of healthcare professionals, medicines, diagnostic equipment, or basic infrastructure remain substantial, introducing sophisticated AI without addressing foundational constraints could increase rather than reduce inequalities.

### 5.7 From Technology Procurement to Health‐System Transformation

The broader lesson from digital‐health implementation is that successful scale‐up is a health‐system transformation process rather than a technology procurement exercise [47, 64, 65]. Sustainable AI adoption requires alignment with national health priorities, institutional ownership, interoperability, financing, local technical capacity, workforce engagement, community participation, and continuous evaluation [60, 63]. Accordingly, the value of AI in Cambodia should not be judged by the number of algorithms developed or pilot projects implemented. Its contribution should instead be evaluated according to whether applications are locally validated, clinically useful, economically sustainable, ethically governed, integrated into routine care, and associated with measurable improvements in health outcomes and equity [42, 43, 58].

## 6 Future Directions and Recommendations

The responsible integration of AI into Cambodia's healthcare system will require coordinated national planning and sustained collaboration across government, healthcare institutions, academia, technology developers, and communities. Several priorities emerge from the current evidence.

‐First, strengthen the digital foundations of healthcare. Reliable connectivity, electricity, interoperable electronic health information systems, secure data infrastructure, and appropriate technical support are prerequisites for sustainable AI deployment, particularly in rural and underserved areas. Digital investments should prioritize interoperability and integration rather than the creation of disconnected platforms.
‐Second, establish a comprehensive governance and regulatory environment. Cambodia should progressively develop clear requirements for data protection, cybersecurity, ethical review, clinical validation, transparency, accountability, procurement, and post‐deployment monitoring. Governance should be sufficiently flexible to accommodate technological innovation while maintaining patient safety and public trust.
‐Third, invest in workforce development and AI literacy. Healthcare professionals require practical competencies to understand AI capabilities, limitations, risks, and appropriate use. AI and digital‐health competencies should be incorporated into continuing professional development and, where appropriate, medical and health‐science curricula. Training should emphasize that AI supports clinical reasoning rather than replacing professional responsibility.
‐Fourth, prioritize local research, validation, and implementation science. Evidence generated in high‐income countries cannot automatically establish effectiveness in Cambodia. Locally conducted studies should evaluate diagnostic and clinical performance, usability, equity, cost‐effectiveness, workflow integration, patient outcomes, and sustainability. Prospective evaluation and post‐deployment monitoring should become standard components of AI implementation.
‐Fifth, evaluate AI according to health‐system value rather than technological novelty. Priority should be given to applications addressing clearly defined health needs and for which sufficient data, implementation capacity, and pathways for evaluation exist. Economic evaluation should consider not only direct costs but also opportunity costs, maintenance requirements, and the sustainability of financing after external project support ends.
‐Sixth, strengthen multisectoral and international collaboration. Partnerships among government agencies, universities, healthcare institutions, technology developers, and international organizations can support knowledge exchange, infrastructure development, research capacity, and responsible technology transfer. Such partnerships should prioritize local ownership and capacity development rather than long‐term dependence on external technical support.
‐Seventh, engage healthcare professionals, patients, and communities in AI development. User participation is essential for ensuring that technologies respond to real clinical and community needs. Transparent communication about the benefits, limitations, privacy implications, and appropriate use of AI can strengthen trust and reduce unrealistic expectations.
‐Finally, AI development should remain patient‐centered, equitable, and human‐led. AI systems should augment healthcare professionals while preserving human oversight, accountability, and patient autonomy. As Cambodia's digital‐health infrastructure matures, AI may support a gradual transition toward more predictive, preventive, and personalized healthcare. However, such progress should be guided by evidence and health‐system priorities rather than technology adoption alone.

## 7 Conclusion

AI presents opportunities to strengthen healthcare in Cambodia by supporting disease diagnosis, clinical decision‐making, telemedicine, public health surveillance, remote monitoring, and health‐system management. Its potential value is particularly relevant to persistent challenges involving geographic disparities in access, limited specialist capacity, increasing chronic disease burdens, and resource constraints. However, the transition from technological potential to measurable health benefit is not automatic. Cambodia's ability to benefit from healthcare AI will depend on the maturity of its digital infrastructure, quality and representativeness of health data, workforce capacity, interoperability, affordability, cybersecurity, governance, and ability to conduct rigorous local validation. The experience of existing digital‐health initiatives also indicates that technical feasibility alone does not guarantee sustainable integration or population‐level impact.

A phased approach that combines digital‐health strengthening, local research, economic evaluation, workforce development, ethical governance, and prospective clinical and public health evaluation is therefore warranted. AI should complement rather than replace healthcare professionals and should be implemented in ways that preserve patient safety, equity, accountability, and human‐centered care. Ultimately, the relevance of AI to Cambodia should be measured not by the sophistication of the technology itself, but by whether it can be responsibly integrated into the health system and produce demonstrable, equitable, affordable, and sustainable improvements in healthcare access and patient outcomes.

## Author Contributions

**Sokchan Lorn**: conceptualization, data curation, formal analysis, validation, writing – original draft, writing – review and editing. **Yanvary Chhon**: validation, writing – review and editing. **Techly San**: validation, writing – review and editing. **Virak Sorn**: conceptualization, data curation, formal analysis, supervision, writing – original draft, writing – review and editing.

## Funding

The authors have nothing to report.

## Ethics Statement

The authors have nothing to report.

## Conflicts of Interest

The authors declare no conflicts of interest.

## Transparency Statement

The manuscript guarantor, Virak Sorn, affirms that this manuscript is an honest, accurate, and transparent account of the study being reported; that no important aspects of the study have been omitted; and that any discrepancies from the study as planned (and, if relevant, registered) have been explained.

## Data Availability

Due to the fact that no data sets were created or examined for this article, data sharing is not applicable.
